# Orbital Myeloid Sarcoma Masquerading as Orbital Cellulitis: A Diagnostic Challenge in an Adult Patient

**DOI:** 10.7759/cureus.110186

**Published:** 2026-06-03

**Authors:** Rahisha Maskey, Sharan Thapa, Oliver W Spencer, Zoe Avila

**Affiliations:** 1 Department of Emergency, Croydon University Hospital, Croydon, GBR; 2 Department of Emergency, Croydon Health Services NHS Trust, London, GBR; 3 Department of Cellular Pathology, St George's Hospital, London, GBR

**Keywords:** acute myeloid leukemia (aml), atypical manifestations of aml, combined chemotherapy, extra-medullary myeloma, intra-orbital mass, ocular chloroma, ocular proptosis, orbital myeloid sarcoma, preseptal orbital cellulitis, runx1::runxt1

## Abstract

Myeloid sarcoma (MS), also known as granulocytic sarcoma or chloroma, is a rare extramedullary manifestation of acute myeloid leukaemia (AML). It represents a tumour-like accumulation of myeloid blasts outside the bone marrow. Orbital involvement is uncommon and may mimic inflammatory or infectious conditions, creating significant diagnostic challenges.

We report a 24-year-old female who presented with a two-week history of progressive left orbital pain and swelling, initially managed as presumed orbital cellulitis with oral flucloxacillin and topical chloramphenicol, which subsequently progressed to worsening swelling, eye pain, and proptosis despite antibiotic therapy, prompting further investigation. Orbital imaging demonstrated a retro-orbital mass compressing the optic nerve. Peripheral blood film demonstrated circulating blast cells, and subsequent orbital biopsy confirmed MS. Cytogenetic analysis revealed t(8;21)(q22;q22), RUNX1-RUNX1T1 fusion, along with FLT3-ITD and KIT mutations, confirming AML.

Following confirmation of the diagnosis, the patient received induction chemotherapy using the DA60 regimen (daunorubicin and cytarabine) combined with gemtuzumab ozogamicin (Mylotarg) as per institutional protocol. Following achievement of remission, consolidation chemotherapy was delivered with two cycles of high-dose cytarabine (HiDAC) in combination with midostaurin, administered via continuous ambulatory drug delivery (CADD) during the second consolidation cycle. Midostaurin was subsequently continued as maintenance monotherapy in view of the confirmed FLT3-ITD mutation. Adjuvant orbital radiotherapy was delivered to a total dose of 25 Gy in 12 fractions. Visual acuity recovered from a baseline of 6/24 to 6/6 at follow-up, with complete resolution of proptosis. The first complete morphological remission was confirmed approximately four weeks after commencement of induction chemotherapy, with serially declining minimal residual disease (MRD) levels on PCR monitoring.

This case highlights the importance of maintaining a high index of suspicion for malignant causes in atypical or treatment-resistant orbital lesions. Orbital MS can closely resemble orbital cellulitis or idiopathic orbital inflammation, delaying accurate diagnosis. Early biopsy and haematological workup are essential for timely intervention. Prompt initiation of AML-directed therapy and multidisciplinary management can significantly improve both visual and systemic outcomes.

## Introduction

Myeloid sarcoma (MS) is a unique form of acute myeloid leukaemia (AML) characterised by a tumour-like proliferation of blast cells occurring outside the bone marrow [1]. Isolated MS is rare, with an estimated incidence of approximately two cases per million adults per year, and demonstrates considerable heterogeneity in clinical presentation, with the existing literature largely limited to case reports and small retrospective series [2]. Orbital involvement is particularly uncommon, accounting for a small minority of all extramedullary presentations, and only a limited number of adult cases have been documented [2,3].

The rapid proliferation of myeloid blast cells within the orbital space produces an acute inflammatory response characterised by periorbital oedema, chemosis, and proptosis, closely mimicking the clinical signs of orbital cellulitis or idiopathic orbital inflammation. This pathophysiological overlap is the primary reason for diagnostic delay in orbital MS. The clinical course of MS is variable; it may present de novo, precede systemic leukaemia, or occur concurrently with or following a diagnosis of AML, further complicating clinical recognition.

In this report, we present a case of orbital MS in an adult initially mimicking orbital cellulitis and review the literature on this presentation, which poses significant diagnostic challenges and may lead to delays in treatment.

## Case presentation

A 24-year-old Afro-Caribbean female presented to our Emergency Department with a two-week history of progressively worsening swelling of the left orbit, associated with ipsilateral lacrimation, photophobia, reduced visual acuity, temporal headache, nausea, and a single episode of vomiting. The condition had initially manifested as a small painless bump on the left eyelid, which subsequently enlarged rapidly to involve the entire eye. Four days before presenting to the Emergency Department, she was reviewed by her general practitioner and prescribed oral flucloxacillin for a presumed infection; however, there was no clinical improvement. She subsequently attended another Emergency Department, where she was discharged with topical chloramphenicol. Her symptoms continued to worsen over the following 48 hours, prompting presentation to our institution for further evaluation.

On examination, her left visual acuity was reduced to 6/24. There was marked proptosis with anterior displacement of the globe, pronounced conjunctival chemosis, excessive lacrimation, and restricted extraocular movements in all directions of gaze. The left periorbital region was tender to palpation (Figure 1). Pupillary examination demonstrated a round left pupil with a relatively sluggish light reflex compared with the right; no relative afferent pupillary defect (RAPD) was elicited. Intraocular pressure in the left eye was 17 mmHg. Slit-lamp examination revealed no fluorescein staining, and there was no corneal exposure or keratopathy. Fundoscopic examination demonstrated normal disc margins bilaterally, with no evidence of disc swelling or pallor. Systemic examination revealed no abnormalities, and vital signs were within normal limits.

**Figure 1:**
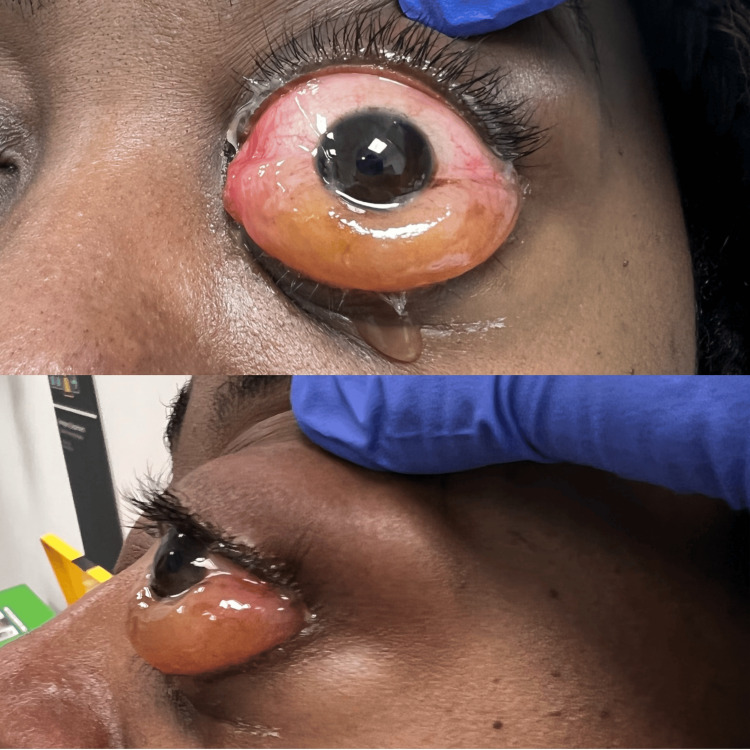
Clinical photograph at presentation demonstrating marked left-sided proptosis with anterior displacement of the globe, pronounced conjunctival chemosis, excessive lacrimation, and restricted extraocular movements.

Initial investigations included a full blood count and peripheral blood film, which demonstrated leukopenia with a white cell count of 2.8 × 10⁹/L and an absolute neutrophil count of 0.7 × 10⁹/L, below the reference range and consistent with neutropenia. Notably, the peripheral blood film also revealed circulating blast cells, raising concern for an underlying haematological malignancy (Table 1). Bone marrow aspirate and trephine (BMAT) performed subsequently demonstrated 64% blasts on morphology and 50% on flow cytometry, with two distinct blast populations identified on both assessments, consistent with AML.

**Table 1:** Initial haematological investigations at presentation, with results and reference ranges. ↓ denotes values below the reference range. * denotes that the peripheral blood blast percentage was not formally quantified on the peripheral blood film; BMAT performed subsequently demonstrated 64% blasts on morphology and 50% on flow cytometry, with two distinct blast populations. BMAT: Bone Marrow Aspirate and Trephine

Parameters	Results	Reference Ranges
Haemoglobin	127 g/L	115-165 g/L
Red cell count	4.11 × 10¹²/L	3.9-5.8 × 10¹²/L
Haematocrit	0.37 L/L	0.37-0.47 L/L
Mean cell volume (MCV)	89.1 fL	78-97 fL
Mean cell haemoglobin (MCH)	31 pg	27-34 pg
Mean cell haemoglobin concentration (MCHC)	348 g/L	310-360 g/L
Red blood cell distribution width (RDW)	13.1%	11.5-16%
Platelet count	328 × 10⁹/L	150-450 × 10⁹/L
White cell count (WCC)	2.8 × 10⁹/L ↓	4-11 × 10⁹/L
Absolute neutrophil count	0.7 × 10⁹/L ↓	1.5-8 × 10⁹/L
Absolute lymphocyte count	1.7 × 10⁹/L	1.1-4.0 × 10⁹/L
Absolute monocyte count	0.4 × 10⁹/L	0.2-1.1 × 10⁹/L
Eosinophil count	0.00 × 10⁹/L	0.1-0.4 × 10⁹/L
Absolute basophil count	0.00 × 10⁹/L	0.0-0.3 × 10⁹/L
INR	1.2	0.8-1.1 ratio
Activated partial thromboplastin time (APTT)	0.94	0.85-1.15 ratio
Clauss fibrinogen level	3.4 g/L	1.6-4.8 g/L
Peripheral blood film	Circulating blasts present *	

Computed tomography (CT) of the orbits (Figure 2), performed at our institution, revealed an enhancing soft-tissue lesion within the superior and lateral retro-orbital region of the left orbit. The lesion extended anteriorly, displacing the optic canal and causing significant proptosis without evidence of intraorbital haemorrhage. The radiological differential diagnosis included an orbital abscess or a neoplastic process such as lymphoma, and further evaluation with magnetic resonance imaging (MRI) was recommended.

**Figure 2:**
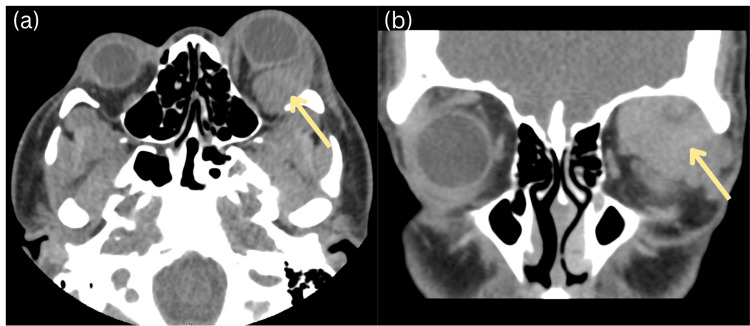
(a)-(b) Axial CT scan of the orbits demonstrating an enhancing soft-tissue lesion within the superior and lateral retro-orbital region of the left orbit, extending anteriorly, displacing the optic canal, and causing proptosis (arrows). CT: Computed Tomography

The patient was subsequently transferred to a tertiary referral centre, where an MRI (Figure 3) of the left orbit demonstrated an enhancing intraorbital mass measuring approximately 4.9 × 3.4 cm (anteroposterior × craniocaudal). The lesion exhibited intermediate T2-weighted signal intensity and restricted diffusion, traversing both intra- and extraconal compartments with involvement of preseptal and postseptal soft tissues. The mass resulted in crowding at the orbital apex and significant compression of the globe, with medial displacement and thinning of the optic nerve, placing vision at substantial risk.

**Figure 3:**
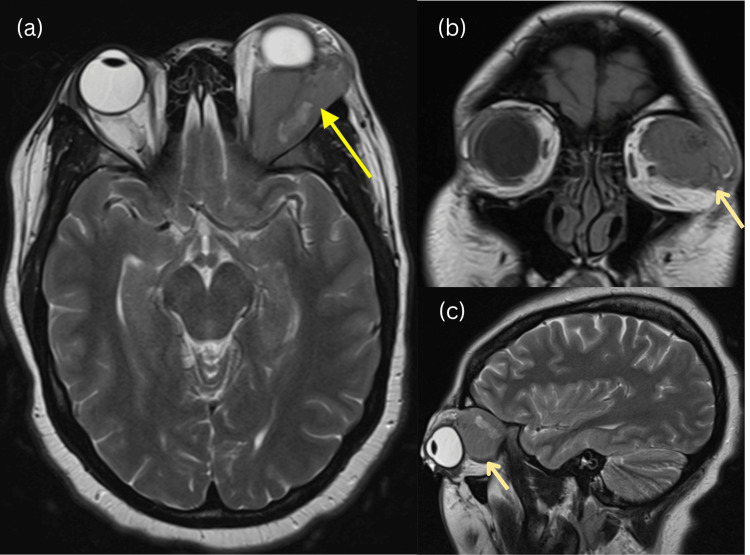
T2-weighted magnetic resonance imaging (MRI) of the orbit and brain in axial (a), coronal (b), and sagittal (c) views demonstrating an enhancing intraorbital mass extending across the intra- and extraconal compartments with pre- and postseptal involvement. The lesion causes orbital apex crowding, significant globe compression, and medial displacement of the optic nerve (yellow arrows).

The patient underwent a left anterior orbitotomy, during which an orbital biopsy and bone marrow biopsy were obtained. Histopathological examination (Figures 4a-4b) revealed infiltration by blasts consistent with AML, where fragments of fibroadipose tissue were extensively infiltrated by a densely cellular basophilic tumour, resulting in complete effacement of the normal architecture. The infiltrate comprised diffuse sheets of atypical round blue cells with foci of apoptosis and necrosis. At higher magnification, the tumour cells exhibited pleomorphic, medium-sized, round-to-oval nuclei with occasional indentations, inconspicuous nucleoli, scant cytoplasm, and frequent mitotic figures.

**Figure 4:**
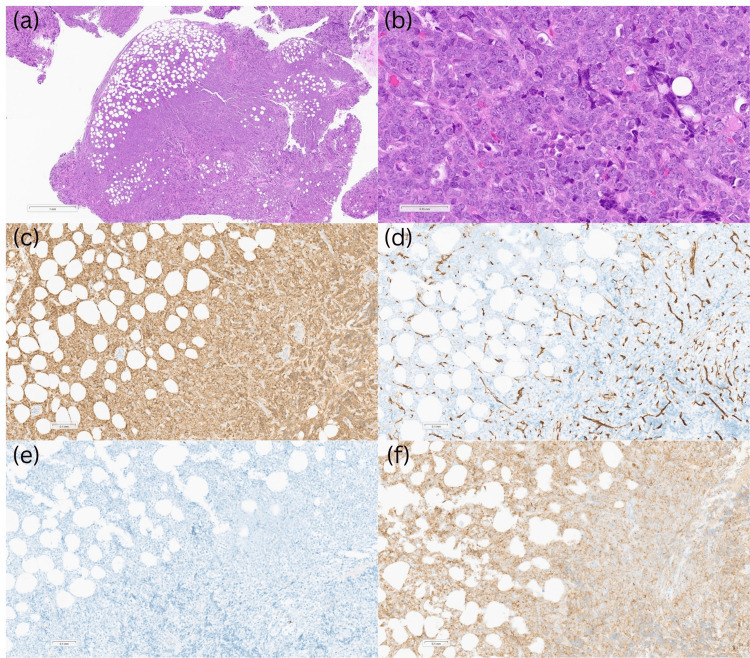
Histopathological examination. (a) Tumour mass comprising fibroadipose tissue extensively and densely infiltrated by a basophilic neoplasm with effacement of the normal architecture (areas of crush artefact present) (H&E stain, 2×). (b) The atypical cells exhibit pleomorphic, medium-sized, round-to-oval, occasionally indented nuclei, with small, indistinct nucleoli and minimal cytoplasm. Mitotic figures are readily identified (H&E stain, 40×). (c) Lesional cells are diffusely positive for myeloperoxidase (MPO) by immunohistochemistry. (d) Lesional cells are negative for CD34 (internal positive control highlights background vasculature). (e) Lesional cells are negative for TdT (positive external control shown). (f) Lesional cells are diffusely positive for CD117 by immunohistochemistry.

Immunohistochemistry (Figures 4c-4f) demonstrated diffuse, strong positivity of the lesional cells for CD4, CD45, CD117, and myeloperoxidase (MPO), supporting myeloid lineage differentiation. The tumour cells were negative for CD34 and TdT, with internal and external positive controls confirming staining adequacy. Additional negative immunostains included AE1/3 and CK8/18 (excluding carcinoma), Sox-10 (excluding melanoma), CD20, CD79a, and Pax-5 (excluding B-cell neoplasms), CD3, CD5, and CD8 (excluding T-cell neoplasms), and CD123 (excluding plasmacytoid dendritic cell neoplasm). The combined histomorphological and immunohistochemical profile was consistent with MS. Although CD34 negativity can occur in extramedullary sites, strong CD117 and MPO expression further supported the diagnosis.

Cytogenetic and molecular analysis demonstrated the presence of t(8;21)(q22;q22), RUNX1-RUNX1T1 fusion, FLT3 internal tandem duplication (FLT3-ITD), and KIT mutations - genetic aberrations commonly associated with AML, each carrying distinct prognostic implications. The presence of the RUNX1-RUNX1T1 translocation is typically associated with a favourable prognosis; however, coexisting FLT3-ITD and KIT mutations may indicate a higher risk of relapse and a more aggressive disease course. These findings confirmed the diagnosis of orbital MS secondary to AML.

The case was discussed at a formal site-specific multidisciplinary team (MDT) meeting, with subsequent review at the Teenagers and Young Adults Cancer (TYAC) MDT in view of the patient's age. Fertility counselling was provided, and egg cryopreservation was completed prior to commencement of chemotherapy.

Induction chemotherapy was commenced with the DA60 regimen, comprising daunorubicin and cytarabine administered over the first 10 days of the cycle, in combination with gemtuzumab ozogamicin (Mylotarg) on Day 1, as per institutional protocol. Following achievement of remission, consolidation chemotherapy was delivered with two cycles of high-dose cytarabine (HiDAC) in combination with midostaurin, administered via continuous ambulatory drug delivery (CADD) during the second consolidation cycle. Midostaurin was subsequently continued as maintenance monotherapy in view of the confirmed FLT3-ITD mutation. Adjuvant orbital radiotherapy was delivered to a total dose of 25 Gy in 12 fractions. Supportive therapy included aciclovir 400 mg three times daily, co-trimoxazole prophylaxis, and gelclair oromucosal gel for mucositis management.

Following completion of combined chemoradiotherapy, visual acuity improved from 6/24 at presentation to 6/36 during the acute treatment phase, with subsequent complete recovery to 6/6 at follow-up. There was complete clinical resolution of proptosis, conjunctival chemosis, and restriction of extraocular movements. The patient achieved the first complete morphological remission approximately four weeks after commencement of induction chemotherapy, with no evidence of residual disease on immunophenotyping. Serial MRD monitoring demonstrated a declining trajectory: PCR quantification of 174 copies per 10⁵ ABL copies at an early post-remission timepoint, reducing to 0.03% (32 copies per 10,000) prior to the fourth treatment cycle at approximately five months from diagnosis. The patient has remained under joint haematological and ophthalmological follow-up, with the most recent review documented at approximately three years from initial diagnosis.

## Discussion

Orbital MS, also known historically as granulocytic sarcoma or chloroma, is a rare extramedullary tumour composed of immature myeloid cells. Although it is commonly associated with AML, it is also seen in other myeloproliferative disorders [1,2].

Orbital MS is more prevalent in children; however, adult cases are increasingly recognised [3]. One of the largest reviews of orbital MS, a 2014 case series from the L.V. Prasad Eye Institute in India, found that 8 of 31 (25.8%) orbital MS cases occurred in adults [3]. Its clinical presentation includes rapidly progressive periorbital swelling, proptosis, pain, and occasionally visual impairment [4-6]. Most of these symptoms overlap with more common orbital pathologies such as cellulitis, lymphoma, or idiopathic orbital inflammation [7]. In this case, the key clues warranting further investigation for a malignant process were the patient’s lack of response to initial antibiotic therapy and the presence of a friable orbital mass on surgical exploration.

Diagnosis in adults is often delayed due to the low prevalence of orbital MS and the overlap of its clinical and radiological features with other orbital tumours. Definitive diagnosis can only be made on histopathological examination and immunohistochemical staining for myeloid markers such as MPO, CD68, and CD33 owing to non-specific and overlapping appearances on imaging modalities [8].

Orbital MS may present de novo, precede, or occur concurrently with systemic leukaemia, most commonly AML [9,10]. The orbital mass is sometimes the first sign that leads to the diagnosis of the underlying AML, as in our case. This case highlights the importance of a complete haematological workup for early diagnosis and treatment, which significantly impacts management and prognosis.

The cytogenetic profile identified in this case warrants specific discussion. The t(8;21)(q22;q22) RUNX1-RUNX1T1 translocation is among the most common recurrent abnormalities in AML and is generally associated with a favourable prognosis under standard induction chemotherapy. However, as demonstrated in our patient, concomitant FLT3-ITD and KIT mutations are recognised to confer a significantly higher risk of relapse and may attenuate the prognostic benefit of the core-binding factor translocation. Published series on orbital MS with t(8;21) have noted a particular predilection for extramedullary disease, which may explain the prominent orbital manifestation in this case. The presence of the FLT3-ITD mutation provided the rationale for incorporating midostaurin - a multi-targeted kinase inhibitor - into the maintenance regimen over 12 cycles of 28 days, consistent with its established benefit in FLT3-mutated AML. Furthermore, the use of gemtuzumab ozogamicin was supported by the confirmed core-binding factor translocation and CD33-positive myeloid lineage.

Management of orbital MS requires a multidisciplinary approach [11,12]. This generally entails systemic chemotherapy following AML protocols, with or without adjunctive radiotherapy for local control [13,14]. Surgery is reserved for relieving compressive symptoms [15,16]. In our case, the patient underwent a combination of adjuvant orbital radiotherapy and systemic chemotherapy, delivered within a dedicated TYAC framework, resulting in rapid clinical improvement and restoration of visual function. This aligns with studies suggesting that early and aggressive treatment can lead to favourable outcomes, especially when the diagnosis is made before systemic disease progression. The overall prognosis of orbital MS remains guarded, particularly in adult patients, with survival largely dependent on the initial response to AML-directed therapy and the presence of adverse cytogenetic or molecular features [17,18]. A summary of selected published adult orbital MS cases is presented in Table 2.

**Table 2:** Summary of selected published adult orbital myeloid sarcoma cases. M: Male; F: Female; NR: Not Reported; CT: Computed Tomography; MRI: Magnetic Resonance Imaging; AML: Acute Myeloid Leukaemia; MS: Myeloid Sarcoma; GS: Granulocytic Sarcoma; MPO: Myeloperoxidase; LCA: Leucocyte Common Antigen; MRD: Minimal Residual Disease; BMAT: Bone Marrow Aspirate and Trephine; HiDAC: High-Dose Cytarabine; MIDOST: Midostaurin; CADD: Continuous Ambulatory Drug Delivery; RT: Radiotherapy; VA: Visual Acuity; ED: Emergency Department; MDT: Multidisciplinary Team; TYAC: Teenagers and Young Adults Cancer service; HSCT: Haematopoietic Stem Cell Transplantation

Author/Year	Age/Sex	Initial Clinical Diagnosis	Imaging Findings	Cytogenetics/Molecular Profile	Treatment Strategy	Visual/Clinical Outcome
Lee et al. (2008) [19]	25M	Orbital mass; initially investigated as an inflammatory lesion	CT: large retrobulbar mass, right orbit	t(8;21)(q22;q22); AML-M2; orbital MS preceded systemic AML by 12 months	Incisional biopsy + orbitotomy for decompression; systemic AML-directed chemotherapy commenced on development of pancytopenia	Decreased proptosis post-decompression; AML developed at 12-month follow-up
Payne et al. (2014) [7]	65M	Orbital pseudotumour/inflammatory mass	CT/MRI: right orbital mass with optic canal and superior orbital fissure involvement	Not reported; AML confirmed on pathology; orbital MS as initial AML presentation	Right modified orbitofrontozygomatic craniotomy for decompression and biopsy; systemic AML-directed chemotherapy	Decreased pain and improvement of vision; decreased proptosis and improved extraocular mobility post-operatively
Aggarwal et al. (2014) [3]	Adults (8 of 31 cases; age range NR)	Orbital cellulitis; inflammatory pseudotumour; lymphoma	Orbital mass on CT/MRI; nonspecific features; diagnosis on biopsy only	Variable; AML concomitant in 41.9%; isolated GS in 59.1% developed AML within 3-21 months	Systemic AML-directed chemotherapy ± radiotherapy	Variable; early diagnosis and prompt treatment are associated with a better prognosis
Li et al. (2024) [20]	Case 1: 33 F; Case 2: 23 F	Unilateral proptosis; no systemic AML symptoms	CT/MRI: unilateral orbital mass; Case 1 engulfing the inferior rectus	MPO, CD43, LCA positive; bone marrow normal at diagnosis; cytogenetics NR	Case 1: resection + chemotherapy + radiotherapy + allogeneic HSCT; Case 2: resection + chemotherapy + radiotherapy	Case 1: no recurrence >5 years; Case 2: no recurrence >10 years
Present case 2022	24F	Orbital cellulitis; treated with flucloxacillin then chloramphenicol across two ED attendances	CT: enhancing retro-orbital soft-tissue lesion; MRI: 4.9 × 3.4 cm intraorbital mass with orbital apex crowding and optic nerve compression	t(8;21)(q22;q22); RUNX1-RUNX1T1; FLT3-ITD; KIT mutation; BMAT 64% blasts (morphology), 50% (flow cytometry)	DA60 + gemtuzumab ozogamicin; CYT-HD + MIDOST; CYT-HD + MIDOST(CADD) then MIDOST; adjuvant orbital RT 25 Gy in 12 fractions; TYAC MDT framework	VA 6/24 → 6/6; complete resolution of proptosis; first complete morphological remission ~4 weeks post-induction; MRD declining to 0.03% by cycle 4; remission sustained at ~3 years

## Conclusions

We have described a rare case of orbital MS in an adult, a presentation that can closely mimic common orbital infections, thereby obscuring the underlying malignant aetiology. This case emphasises the importance of early clinical suspicion and prompt tissue diagnosis in treatment-resistant orbital conditions. The favourable outcome in this case was achieved through prompt multidisciplinary management, including AML-directed induction chemotherapy with the DA60 regimen and gemtuzumab ozogamicin, FLT3-targeted therapy with midostaurin in view of the confirmed FLT3-ITD mutation, and adjuvant orbital radiotherapy to a total dose of 25 Gy in 12 fractions. Visual acuity recovered from a baseline of 6/24 to 6/6 at follow-up, with complete resolution of proptosis. First complete morphological remission was confirmed at approximately five weeks following commencement of induction chemotherapy, with serially declining MRD levels demonstrated on PCR monitoring. The patient has remained in remission for approximately three years since the initial diagnosis.

Evidence from this case suggests that timely initiation of aggressive chemoradiotherapy, combined with targeted molecular therapy, can result in rapid and sustained functional recovery; however, long-term prognosis remains strongly related to the underlying haematological condition and the presence of adverse cytogenetic or molecular features. Broader awareness of this uncommon presentation among clinicians can facilitate earlier diagnosis and more favourable outcomes.
